# PCD’s Commitment to Advancing Diversity, Equity, and Inclusion in Its Scientific Leadership, Peer-Review Process, Research Focus, Training, and Continuing Education

**DOI:** 10.5888/pcd18.210269

**Published:** 2021-08-12

**Authors:** Leonard Jack

**Affiliations:** 1 Preventing Chronic Disease, Office of Medicine and Science, National Center for Chronic Disease Prevention and Health Promotion, Centers for Disease Control and Prevention, Atlanta, Georgia

This position statement expresses *Preventing Chronic Disease*’s (PCD’s) commitment to continuously assess our focus, document our accomplishments, and identify new areas of growth. It begins with a brief overview of the burden of chronic diseases in the United States to emphasize why the journal remains committed to publishing peer-reviewed content that contributes new knowledge on innovative ways to ameliorate these long-standing public health challenges. Keeping PCD in the best position to publish relevant peer-reviewed articles requires that we continue our efforts to advance diversity, equity, and inclusion as well as best practices at all levels of operation. Hence, this position statement discusses the evolution of the journal’s mission statement and its current topic areas of interest and proposes activities to advance diversity, equity, and inclusion (DEI) through scientific leadership, the peer-review process, research focus, and provision of training and continuing education.

Chronic diseases such as heart disease, cancer, diabetes, and obesity are among the leading causes of death, are the costliest to treat, and affect one-third of adults worldwide (1). Risk factors such as tobacco use, lack of physical activity, and poor nutrition have long been recognized as primary contributors to chronic disease prevalence and are, thus, the focus of public health efforts (2–12). These risk factors have historically helped to show where to implement public health interventions to address long-standing health disparities. For example, chronic disease prevention and management interventions may focus on behaviors such as healthy eating and physical activity and cessation of unhealthy practices such as tobacco and alcohol use (13–15). But there is also an awareness that these unhealthy behaviors exist within a larger context that goes beyond the individual.

The impact of chronic diseases is disproportionately evident in low-resourced areas and in communities of color (16,17). In these communities, chronic diseases are influenced by a combination of coexisting and interactive factors — race and ethnicity, psychological issues, socioeconomic status, culture and history, access to health care, racial discrimination, and environmental determinants of health (18,19). Furthermore, the COVID-19 pandemic demonstrated that these factors exacerbate chronic disease disparities in diverse communities that are also disproportionately affected by COVID-19. Responding to long-standing health inequities and health disparities requires scientific peer-reviewed journals to play a critical role in widening the scope of their content to acknowledge, explore, and report on less-studied factors, such as social determinants of health. These include forms of racism that have resulted in generational injustices, which also contribute to the rise of racial and ethnic health inequities (20). Broadening the definition of social determinants of health to include the influence of racism enables a better understanding of how these risk factors also affect where people live, learn, work, worship, and play. This expanded area of study can also highlight how racism contributes to inequities in access to a comprehensive range of social and economic benefits — including housing, education, wealth, and employment — that ultimately affect population health.

Since its establishment in 2004, PCD’s mission has been to promote dialogue among researchers, practitioners, and policy makers worldwide on the integration and application of research findings and practical experience to address health disparities, advance health equity, and improve population health. We recognize that both the journal and the field of public health cannot effectively achieve this mission by doing things the same way and expecting different results; success requires an improved understanding of the factors that shape health along with knowing where, how, and when to intervene effectively. To be successful in this mission requires that PCD and other peer-reviewed journals adapt to a changing vocabulary and embrace areas of scientific exploration to include not only familiar terms and constructs, such as race and ethnicity, health disparities, health inequities, social economic position, and social determinants of health, but also all forms of racism, including structural and institutional racism.

PCD is well positioned to address chronic disease prevention and health promotion in this changing landscape. Over the past 5 years, we have taken intentional steps to identify ways to increase the participation of talented, experienced, and well-trained researchers, evaluators, policy makers, and practitioners who bring attention to new issues in the published literature. We took these steps long before DEI began receiving increased attention in scientific publishing. We took steps to ensure *diversity* among volunteers serving in every major group that provides feedback to the journal — external review panels, associate editors, editorial board members, Statistics Review Committee members, guest editors, peer reviewers, and PCD staff members. In doing so, we have consciously worked against unintentional promotion of one view or perspective at the exclusion of others, which can result in disengaging individuals and reducing participation among key players and community partners.

PCD continues to advance *equity* by proactively listening and then implementing feedback, recognizing the contributions of all volunteers, and providing a range of opportunities for others to lead and participate in key decision making. Our success in these areas is the result of creating an open dialogue among various partners — primarily those external to the Centers for Disease Control and Prevention — to generate the journal’s mission and vision statements, identify topic areas for calls for papers, serve as guest editors of supplemental collections, refine the journal’s peer-review processes, develop manuscript guidance documents, secure specialized peer reviewers, and more.

To ensure *inclusion*, we took proactive steps so that a range of individuals are and will continue to be part of discussions that help us present a broad spectrum of ideas and perspectives. PCD’s purpose here is to prevent any one paradigm, belief, or perspective in the science and practice of public health to dominate decision making. We want to ensure that the journal’s content areas help to advance the most comprehensive understanding of the range of both causes and solutions to long-standing public health challenges. For example, in 2017, we invited the journal’s first panel of 7 nationally recognized experts (Appendix) in scientific publishing, population health, epidemiology and surveillance, social epidemiology, community health, health disparities, health equity, medicine, and community health to critique our focus, mission, publication content, and intended audience and offer recommendations on future directions (21). Based on recommendations from the expert panel, we decided to complement our publication of epidemiological studies with increased attention to securing manuscripts from researchers, evaluators, policy makers, and practitioners working in settings that improve health through population-based interventions and policies. The panel felt the journal had been in existence long enough to revise and expand its key areas to focus on 4 main topics:

- Behavioral, psychological, genetic, environmental, biological, and social factors that influence health
- Development, implementation, and evaluation of population-based interventions to prevent chronic diseases and control their effect on quality of life, illness, and death
- Interventions that reduce the disproportionate incidence of chronic diseases among populations at high risk of developing these diseases
- Development, implementation, and evaluation of public health law and health policy–driven interventions

Expanding the journal’s focus beyond articles on behavioral, psychological, genetic, environmental, biological, and social factors required securing volunteers with expertise in areas such as identifying and tracking disease prevalence; cultural identity; community engagement; health behaviors; health disparities; minority health; sexual orientation; lesbian, gay, bisexual, transgender, or queer (LGBTQ) health; health equity research and practice; implementation science; multilevel interventions and data analyses; structural and environmental supports; policy changes; geospatial epidemiology; health system changes; and others. We have taken deliberate and timely steps to secure individuals at different career stages with diverse racial and ethnic backgrounds, gender identities, geographic locations, training, and experiences to serve as associate editors and on our editorial board and our Statistics Review Committee. Please visit the journal’s website to learn more about these individuals who volunteer their time and expertise to advance the journal’s mission and vision (https://www.cdc.gov/pcd/about_the_journal/index.htm).

PCD will build on past efforts to advance DEI by securing and maintaining scientific leadership that consists of skilled, trained, respected, and courageous volunteers who are not afraid to voice their opinions to ensure that the journal is relevant and responsive to advances in the science and practice of public health. We look forward to learning from our volunteers how public health research and evaluation can examine less-explored ways of conducting health disparities research, by taking a fresh look at traditional determinants of health and advancing health equity to better define constructs around race and the impact of racism — broadly defined — given that this determinant is a serious threat to the public’s health (22,23).

By December 2021, we will finalize our strategic plan to advance DEI activities through continued expansion of 4 key areas:

- Scientific leadership
- The peer-review process, including enlarging the pool of authors and peer reviewers
- Expanding research to identify potentially effective ways to improve health equity and shed light on the intersection of racism and health
- DEI-related training and continuing education opportunities among PCD staff members, volunteers, peer reviewers, and authors

We believe these activities, when undertaken collectively, will help the journal continue to serve as a critical resource of relevant and responsive peer-reviewed content focused on eliminating health disparities and advancing health equity (Figure).

**Figure:**
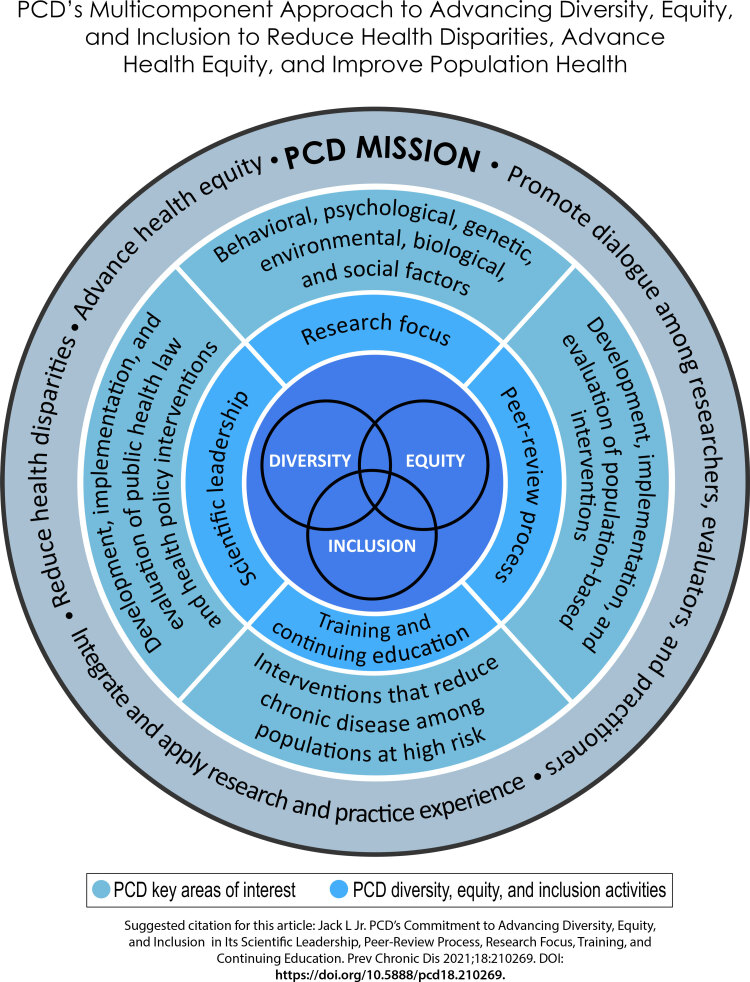
*Preventing Chronic Disease*’s multicomponent approach to advancing diversity, equity, and inclusion to reduce health disparities, advance health equity, and improve population health.

## Continuing Efforts to Expand PCD Scientific Leadership

We will continue conversations with our associate editors, editorial board, Statistics Review Committee members, and other scholars and experts to identify ways to advance DEI in several key areas. Despite our previous success with securing diversity in expertise, racial and ethnic background, geographic location, institution, career status, and other areas, we will continue to assess our needs and proactively work to obtain additional volunteers in these key roles. We will continue discussions with our volunteers on ways to advance DEI at all levels of journal operations: in appointing board and committee members, in widening the scope of topic areas, in considering the impact of all forms of racism on health, and in increasing awareness through minority-serving institutions, organizations, and networks to encourage submissions from researchers from diverse races and ethnicities. We will continue to assess needs and make appointments annually to these groups that are vital to the journal’s operations.

PCD is also committed to giving authors as many tools as possible to improve the quality of manuscripts submitted for consideration. The Author’s Corner section of the PCD website provides detailed guidance on how to generate a manuscript for submission. In addition, we expanded our article types to increase the number of submissions that advance our understanding of how multiple, competing, and interconnected determinants shape health; how aspects of the environment (including setting and location) and diverse community partners must be considered to create viable solutions to improving conditions that influence health; and how the discovery of new and improved ways to capture data and report findings make it possible to learn what is working. PCD is also committed to identifying factors that influence dissemination and uptake of innovative and effective policy-level interventions. We will continue to update this guidance to encourage integrating, where possible and necessary, information on the impact of racism on a range of health outcomes and potential ways to advance health equity. Prospective authors can learn more about the simplified submission process at https://www.cdc.gov/pcd/for_authors/SimplifiedSubmissionProcess.htm and see criteria and descriptions of each PCD article type at https://www.cdc.gov/pcd/for_authors/types_of_articles.htm. This guidance has been a valuable resource to authors in generating the best manuscripts for submission, and it is used by peer reviewers to evaluate submissions and recommend their suitability for publication.

## Peer-Review Process: Expanding Authorship and Peer Reviewers

PCD has done an exceptional job securing contributions from authors around the world. However, like other peer-reviewed journals, we have an opportunity to encourage less-represented individuals and groups — inclusive of gender identity, sexual orientation, race and ethnicity, age, disability status, socioeconomic status, geographic location, and institution — to submit manuscripts for consideration. We will continue our efforts to identify potential contributing authors to submit manuscripts related to the journal’s 4 focus areas and any special topics through our many calls for papers. PCD will also continue to identify and recruit people from these groups to serve as guest editors and reviewers on manuscripts submitted in response to calls for papers and general submissions to the journal. PCD will also continue to provide feedback to authors, when necessary, on ways to strengthen their submissions. We will identify, with the assistance of our editorial board members and associate editors, ways to provide resources on the PCD website that further develop scientific writing skills among novice authors. For example, PCD’s identification of best practices in scientific writing, along with those identified by other experts, were incorporated into an online scientific training course consisting of 8 modules that addressed topics ranging from basic writing principles to abstracts to components of a research report (introduction, methods, results, discussion), to supporting materials, and finally to submitting the manuscript for publication. This online training course will be available on the journal’s website in English and Spanish in early 2022.

## Research Focus: Expanding Research and Evaluation Topic Areas of Interest

PCD’s topic areas during 17 years of publication have evolved and expanded to address multiple pressing public health issues (24), including the following:

- Understanding causes of health disparities and how such discoveries can be translated into evidence-based interventions to address them
- Using implementation science to understand the ways in which evidence-based interventions are adopted — including exposure, dose, quality of delivery, participant responsiveness, and program differentiation — in real-world settings
- Developing and applying spatial statistical methods and new geospatial tools to identify and intervene on drivers that affect health at multiple geographic levels
- Using maps and geospatial results to guide program and policy decision making
- Promoting health and wellness among diverse racial and ethnic groups, socioeconomic and educational levels, and geographic locations
- Implementing risk communication approaches through preparation, response, and recovery phases of major health threats
- Improving population health through collaboration between public health and pharmacy
- Reporting on public health responses to COVID-19 and chronic disease
- Collecting and using surveillance data to inform policy changes, guide new program interventions and public communications, and assess research investments
- Evolving population health approaches to address mental health
- Sustaining changes in how health care systems, public health, and other sectors address social determinants of health in partnership with community-based organizations
- Identifying better and best population health practices to improve population health across the lifespan
- Developing, implementing, and evaluating public health law and health policy–driven interventions

The articles published by PCD on these topics represent a collaborative effort. Although PCD is an editorially independent journal, we are continually engaged with peer reviewers, associate editors, editorial board members, Statistics Review Committee members, external panels, and other experts who provide critical input and feedback on important issues facing public health from their position of expertise. We value engagement as part of the dialogue that needs to happen to better understand the complexity of the landscape of chronic disease prevention. And as an integral part of this dialogue over the years, we recognize a simple truth: no single area of focus can or will provide the solution to ameliorating long-standing public health challenges in chronic disease prevention and control. Instead, a combination of these approaches, inclusive partnerships, patience, commitment, and sustainability, along with rigorous research and evaluation, are needed to monitor and document progress. While acknowledging the contributions of previously published articles, we will continue to engage with partners as this dialogue evolves, as new evidence becomes available, and as new areas of research emerge. With that in mind, and based on feedback from our editorial board, associate editors, and Statistics Review Committee members, and in consultation with experts in the field, PCD’s focus of interest will expand this year to include 2 urgent and pressing issues in public health: identifying potentially effective ways to improve health equity and exploring the intersection between racism and health.

### Where, when, and how to effectively intervene to improve health equity

Advancing health equity and eliminating health disparities have been and continue to be critical areas of great interest to us. Healthy People 2020 defines health equity as the attainment of the highest level of health for all people: “Achieving health equity requires valuing everyone equally with focused and ongoing societal efforts to address avoidable inequalities, historical and contemporary injustices, and the elimination of health and health care disparities” (25). Healthy People 2020 defines health disparities as “a particular type of health difference that is closely linked with social, economic, and/or environmental disadvantage” (25). We want to understand where, when, and how health equity–related interventions should be implemented. Furthermore, we want to learn from the field about the effectiveness of innovative interventions that address the root causes of health inequities. We plan to release a call for papers seeking submissions on the creation of health equity constructs, theories, frameworks, and outcomes to advance the field’s understanding of how to design, implement, and evaluate such interventions. Given that contributing causes of health inequities result from complex underlying and sustained structures, we will be interested in manuscripts that document how collaborations across diverse partnerships are used. We anticipate releasing this call for papers and naming guest editors no later than August 27, 2021.

### The intersection of racism and health

A growing body of peer-reviewed literature provides evidence of the generational effects of various forms of racism on educational systems, housing practices, mental health services, and other areas (23). The impact of racism on health status can be examined collectively to better understand causes of downstream health disparities, particularly among racial and ethnic groups who have experienced decades of disproportionately poor health outcomes. Although this downstream effect has at times been acknowledged, it has not been rigorously explored to identify the mechanisms and pathways through which it operates. Clearly, this represents an important emerging area of research, evaluation, and implementation science in chronic disease prevention and control. We will expand our interest in receiving manuscripts that examine forms of racism and their deleterious effects on other social determinants of health, chronic conditions, and overall physical, mental, and emotional health. Specifically, PCD will make widely known our interest in receiving papers that explore the intersectionality of structural racism and other social determinants of health (socioeconomic position, social support, culture, access to health care, residential environment, and access to environments that support active living and healthy eating). A universal way of measuring structural racism does not exist. Hence, PCD will play an important role in increasing knowledge and identifying best methodologic approaches to quantify structural racism’s association with, or driver of, physical and mental health inequities (23). We will also promote these topics of interest widely to our authors and peer reviewers. By November 30, 2021, we will engage our editorial board and consult with leading experts in the field to develop and release a call for papers that examine the negative impact of all forms of racism on aspects of chronic disease prevention, management, and control.

In May 2017, the National Institute on Minority Health and Health Disparities and the US Department of Health and Human Services Office of Minority Health conducted a workshop to identify and determine ways to incorporate constructs of structural racism and discrimination into health and health disparities research (26). We will use findings from this workshop along with input from our associate editors, editorial board, and Statistics Review Committee members; results from emerging peer-reviewed literature; and consultations with leading experts to identify a range of topics of interest to the journal. These future topic areas will be shared on the journal’s homepage, integrated into author checklists for all article types, and incorporated, where appropriate, in calls for papers. In February 2021, PCD released “COVID-19 and Chronic Diseases: Burden, Access to Care, Community Engagement, and Partnerships,” a call for papers that recognizes that persistent social determinants compound the negative effects of COVID-19 on people with a chronic condition. We are interested in all article types (eg, essays, original research, program evaluation, systematic reviews, tools for public health practice, implementation evaluation) to help further elucidate factors (eg, unstable housing, racism, limited access to nutritious food, inadequate transportation, low socioeconomic status) that affect health outcomes. This call for papers represents the journal’s commitment to increasing rigorous scholarship with an intentional focus on the impact of various forms of racism on health and health disparities. Papers must be submitted to the journal by December 3, 2021. Please visit https://www.cdc.gov/pcd/announcements.htm for more details.

## Offering DEI Training and Continuing Education

We have identified opportunities to advance our understanding of best DEI practices in scientific publishing, and we will maintain our commitment to seek the participation of contributors regardless of race, ethnicity, gender identity, sexual orientation, disability, religion, age, or geographic location. The journal is better positioned to publish rigorous content that can lead to reducing health disparities and advancing health equity when authors of manuscripts undergoing rigorous peer review reflect the population of those most affected. We will continue to maintain an environment among our staff members, volunteers, authors, and peer reviewers that supports increasing knowledge about DEI best practices. For example, several timely resources, such as Toolkits for Equity, published by the Coalition for Diversity and Inclusion in Scholarly Communications, offer PCD staff members access to training and continuing education opportunities not previously available. PCD’s editorial staff has updated our internal house style to reflect the revised inclusive language section of the *AMA Manual of Style: A Guide for Authors and Editors* (27), and the reporting of race and ethnicity in medical science journals.

In 2016, we began conducting orientations for newly appointed associate editors, editorial board members, and Statistics Review Committee members. This time was used to familiarize appointees about their roles, discuss the journal’s peer-review process and research and evaluation standards, review author submission checklists, learn how to effectively assess feedback from peer reviewers, review the journal’s expectations on scientific integrity, and receive guidance on providing concrete and respectful feedback to authors. All volunteers are required to participate in an orientation designed to position them to achieve optimal success in their roles during their appointment term. We will incorporate PCD’s commitment to advancing DEI best practices in all aspects of our operations. We recognize that advancing DEI best practices will require all involved to share in this responsibility so as not to place greater demands intentionally or unintentionally on any individual or subset of individuals.

This year, PCD celebrates the 10-year anniversary of efforts to build scientific publishing skills and abilities among diverse students. We play an important role in enhancing diversity in the student pipeline as a way of helping to create generational diversity across the field of public health. To date, we have received more than 500 student papers submitted in response to our student paper research contest in the following 5 categories: high school, undergraduate, master’s, doctoral, and postdoctoral (28–30). Specifically, we aim to provide applicants with an opportunity to

- Become familiar with a journal’s manuscript submission requirements and peer-review process
- Connect their knowledge and training on conducting quality research with a journal’s publication expectations
- Develop their research and scientific writing skills to become producers of knowledge in addition to consumers of knowledge
- Be first author on a peer-reviewed article
- Engage in supportive, respectful, and mutually beneficial author–mentor relationships that result in strengthening applicants’ ability to generate and submit future scholarly manuscripts

In June 2021, PCD released a new call for papers for our Student Paper Contest. Students from high school to the postdoctoral level are encouraged to submit manuscripts relevant to the prevention, screening, and surveillance of chronic diseases; population-based interventions for chronic diseases; and social determinants of health as they relate to chronic disease prevention, which will now include the intersection of racism, health, and health disparities. Chronic diseases and public health concerns of greatest interest to the journal include but are not limited to cancer, diabetes, cardiovascular disease, obesity, Alzheimer’s disease, epilepsy, arthritis, oral health, asthma, reproductive health, and the bidirectional relationship between COVID-19 and chronic conditions. Students and their faculty mentors interested in submitting research manuscripts to the journal for consideration must do so no later than March 28, 2022. Please visit https://www.cdc.gov/pcd/announcements.htm for more details on the journal’s Student Paper Contest.

Since April 2013, PCD has offered readers opportunities to earn continuing education credit via Medscape, LLC, a leading organization in professional education and continuing medical education (CME) for health care professionals. Medscape, LLC, is jointly accredited by the Accreditation Council for Continuing Medical Education, the Accreditation Council for Pharmacy Education, and the American Nurses Credentialing Center to provide continuing education for the health care team. In collaboration with Medscape, PCD provides opportunities for health care professionals to earn continuing education credit by reviewing original research articles. We anticipate publishing timely articles exploring the impact of all forms of racism on health and possible evidence-driven interventions to improve health equity. Our readers and registered users of Medscape can take the test and earn credit in a variety of topic areas to include these expanded areas, as well as numerous other timely research topic areas. It is an advantage for readers of the journal because it offers another resource for earning CME credits, and it is an advantage for authors because it gives them the recognition that their article was not only selected for publication in PCD but also considered relevant as an educational resource for researchers, clinicians, and physicians. Our intention to publish articles on racism and health aligns with recently published position statements by major medical associations encouraging their professions and associated health care providers to understand more deeply the less-explored and less-acknowledged causes of health disparities (31–33).

## Conclusion

The call to advance DEI best practices across many levels of a journal’s operations has received increasing attention. PCD is well positioned to put into practice the input from our diverse and inclusive groups of volunteers on advancing these imperatives. This, our journal’s position statement, sets forth our continued commitment to advancing DEI through continued expansion of 4 key areas: 1) the journal’s scientific leadership, 2) the peer-review process, 3) research focus (including the intersection of racism and health and developing, implementing, and evaluating interventions to address health inequities), and 4) DEI training and continuing education. We will finalize our strategic plan to implement these DEI activities by the December 2021.

In February 2017, PCD published its first Editor in Chief’s Column — a column published 2 to 3 times annually to provide updates on the journal’s progress, public health topics, announcements and acknowledgments, and issues of interest to the field of public health and the journal’s readership. We will continue to use our Editor in Chief’s Column to provide updates on the journal’s progress in advancing the 4 DEI activities. The next update on progress is scheduled to be published in an Editor in Chief’s Column in mid-November 2021. As always, PCD is open to receiving feedback through the Contact Us page (https://www.cdc.gov/pcd/contactus.htm), and we look forward to updating you on our progress over the coming months.

## Appendix. *Preventing Chronic Disease* 2017 External Review Panel

**Table:**

	Hector Balcazar, PhD, MS (Chair)
	Hector Balcazar is dean of the College of Science and Health at Charles R. Drew University of Medicine and Science. Dr Balcazar specializes in the study of public health problems of Latinos/Mexican Americans. He has conducted numerous studies of Latino birth outcomes, acculturation and health-related behaviors, cardiovascular disease prevention programs in Latinos, and border health issues. He served as an editorial board member for several journals and served as chair of the editorial board of the *American Journal of Public Health*.
	Ana F. Abraido-Lanza, PhD (Co-Chair)
	Ana Abraido-Lanza is professor of sociomedicine and director of the doctor of public health program at the Mailman School of Public Health at Columbia University. In addition, Dr Abraido-Lanza serves as the director of the Initiative for Minority Student Development. Her research focuses on cultural, psychological, and socioeconomic processes that affect psychological well-being, adjustment to chronic illness, and mortality among Latinos. Dr Abraido-Lanza is an associate editor at the journal *Health Education & Behavior* and a member of the editorial board at the *Annals of Behavioral Medicine*.
	Collins O. Airhihenbuwa, PhD, MPH
	Collins Airhihenbuwa is former professor and dean of the College for Public Health and Social Justice at Saint Louis University (SLU). He was also the director of the Global Health Institute at SLU. Dr Airhihenbuwa is an internationally recognized expert on behavioral health and a pioneer in centralizing culture in health behaviors. He has served on editorial boards for several peer-reviewed journals: the *American Journal of Health Behavior*, *AIDS Education and Prevention*, *Health Education & Behavior*, the *Journal of Health Communication*, and the *Journal of Medical Anthropology*.
	Melissa Grim, PhD
	Melissa Grim serves as chair and professor at Radford University in the Department of Health and Human Performance. Dr Grim’s expertise and interests include planning and evaluating public health interventions to increase physical activity and investigating differences in health behavior in urban, rural, and suburban settings. She currently serves as the deputy editor for *Health Promotion Practice*, a journal dedicated to linking research and practice.
	Shiriki Kumanyika, PhD, MPH
	Shiriki Kumanyika is a research professor in the Dornsife School of Public Health at Drexel University. Dr Kumanyika retains an appointment as an emeritus professor of epidemiology at the University of Pennsylvania. She was vice-chair of the US Department of Health and Human Services’ Secretary’s Advisory Committee on Healthy People 2020 objectives, is a past president of the American Public Health Association and is a member of the National Academy of Medicine (formerly known as the Institute of Medicine). She is currently a member of the CDC Task Force on Community Preventive Services, co-chair of the Policy and Prevention Section of the World Obesity Federation, and a member of *The Lancet* Commission on Obesity.
	William L. Lanier Jr, MD
	William L. Lanier is professor of anesthesiology at the Mayo Clinic. His research interests involve neurosurgical anesthesiology and ischemic brain disease, and he has been engaged in both laboratory and clinical research. He was a founding section editor of the *Journal of Neurosurgical Anesthesiology* and founding editorial board member for *Disaster Medicine and Public Health Preparedness*. Dr Lanier served as a faculty member for the Council of Science Editors (CSE) for its 2-day Short Course for Editors and director of the CSE’s Short Course. He served as editor in chief of *Mayo Clinic Proceedings*, the world’s third-largest circulation scholarly medical journal. He is emeritus editor in chief of *Mayo Clinic Proceedings*.
	Sarah Patrick, PhD, MPH
	Sarah Patrick is director of the Jackson County (Illinois) Department of Health. She directs and manages 3 major divisions of the Department of Public Health: Communicable Disease Control, Health Promotion and Public Health Research, and Environmental Health. Dr Patrick has extensive experience developing and supporting collaborative public health practice partnerships between local health departments, academic institutions, and hospital systems. In 2015, she served on a project led by the Council of State and Territorial Epidemiologists to identify scientific writing needs among applied epidemiologists.

All positions reflect those at the time of appointment to the panel.
